# Mid-Adolescent Predictors of Adult Drinking Levels in Early Adulthood and Gender Differences: Longitudinal Analyses Based on the South Australian School Leavers Study

**DOI:** 10.1155/2016/1489691

**Published:** 2016-08-21

**Authors:** Paul H. Delfabbro, Helen R. Winefield, Anthony H. Winefield, Anne Hammarström

**Affiliations:** ^1^School of Psychology, University of Adelaide, Adelaide, SA 5005, Australia; ^2^Department of Public Health and Clinical Medicine, Umeå University, 901 Umeå, Sweden

## Abstract

There is considerable public health interest in understanding what factors during adolescence predict longer-term drinking patterns in adulthood. The aim of this study was to examine gender differences in the age 15 social and psychological predictors of less healthy drinking patterns in early adulthood. The study investigates the relative importance of internalising problems, other risky health behaviours, and peer relationships after controlling for family background characteristics. A sample of 812 young people who provided complete alcohol consumption data from the age of 15 to 20 years (5 measurement points) were drawn from South Australian secondary schools and given a detailed survey concerning their psychological and social wellbeing. Respondents were classified into two groups based upon a percentile division: those who drank at levels consistently below NHMRC guidelines and those who consistently drank at higher levels. The results showed that poorer age 15 scores on measures of psychological wellbeing including scores on the GHQ-12, self-esteem, and life-satisfaction as well as engagement in health-related behaviours such as smoking or drug-taking were associated with higher drinking levels in early adulthood. The pattern of results was generally similar for both genders. Higher drinking levels were most strongly associated with smoking and marijuana use and poorer psychological wellbeing during adolescence.

## 1. Introduction

In Australia, excessive alcohol consumption has been recognised as a significant public health problem. Current estimates suggest that over 75% of the adult population drinks alcohol at least once per year and that 20% drinks in excess of the recommended National Medical and Health Research Council (NHMRC) guidelines [1]. A higher level of alcohol consumption is recognised as having a number of negative social, economic, and health consequences, with links to increases in public disorder, domestic violence, and poorer long-term health [1]. More recently, it has been argued that even modest consumption of alcohol above the NHMRC guidelines of two standard drinks per day can significantly elevate the long-term risk of developing certain forms of cancer [2]. Although alcohol is known to affect all segments of the population, it is recognised that young people, and most notably males, under the age of 25 appear to be one of the highest risk groups [3]. This population is important for two reasons. Young people are most likely to drink at high levels and to engage in binge drinking [4]. This early period of life also has long-term consequences, in that patterns of behaviour established during this period of time may often carry on into latter life and have health effects that are not evident until some years later [5].

Despite decades of public health campaigns and other government strategies to address youth drinking, excessive alcohol consumption remains an ongoing problem for health authorities. One important reason for this is that drinking is a highly normalised and socially sanctioned behaviour amongst young people. Drinking features heavily in social activities and is considered a symbol of adulthood [4, 6]. As Scholte et al. argue, young people also, because of their immaturity and sense of invulnerability, often engage in higher risk activities because these are endorsed by their peers and are considered exciting or enjoyable. Studies have also shown, however, that there is considerable variability in this behaviour. Not all young people necessarily engage in excessive drinking and many may not continue this behaviour into adulthood. For example, as shown in longitudinal studies particularly from Scandinavia (e.g., [7, 8]), the association between elevated drinking at age of 16 years and subsequent years is often only modest. Given these results, there is interest in identifying those young people who are at the highest risk of drinking excessively when they are younger (i.e., during adolescence) and then continuing this behaviour into early adulthood. Such information would provide public health authorities with a better sense of where to target interventions demographically and what school-level intervention points or strategies might be most useful.

Research of this nature has been conducted for several decades at both a national and international level. Despite some differences in methodology (e.g., duration of follow-up, measure of alcohol consumption, and statistical techniques used), the findings from these studies have tended to converge on a number of consistent conclusions. In general, it is concluded that patterns of drinking in young adults are likely to be influenced by a combination of sociodemographic, social, and psychological factors. In particular, young adults with elevated drinking patterns tend to come from family backgrounds where there is disruption, less parental supervision, or a history of heavier alcohol use (e.g., [9, 10]). Those in this group are also more likely to have peers who drink or who have positive attitudes towards drinking [6, 11]. Other risk factors relate to problems of adjustment, including poorer engagement with school [12], higher scores on indicators of externalising or delinquent behaviour [13, 14] including smoking during adolescence [4], and elevated levels of internalising problems such as depression, anxiety, or low self-esteem [14, 15]. In a review of this literature, Mulder [16] suggests that the strongest predictors are usually conduct disorder and that internalising behaviours/emotional problems are very likely as much a symptom of excessive alcohol use as a cause.

Evidence consistently shows that young men typically consume more alcohol than women. They start drinking at a younger age and are more likely to engage in episodes of binge drinking [3]. However, the evidence in support of gender differences in risk factors has generally been less consistent, very likely due to differences in methodology across different studies [17]. For example, while some studies have suggested that young males are more likely to drink to escape from depression, other studies have found evidence for these relationships in young women. Similarly, although young males tend to score higher on measures of antisocial or externalising behaviours, Nolen-Hoeksema's review suggests that evidence of these behaviours in either gender is related to both alcohol use and the prevalence of alcohol-use disorders. It is noteworthy, however, that this review provides less evidence concerning potential gender differences relating to the importance of familial or social relationships. Although there is some analysis of the evidence relating to the influence of partners, research into adolescent drinking patterns would also usually need to consider the potentially important role played by family background, peer relationships, and school engagement.

From a methodological perspective, much of the literature in this area has converged on the understanding that the effects of alcohol need to be studied over a longer period because of the likely cumulative impact on health and wellbeing. Thus, knowing that a person has established a more consistent pattern of elevated drinking becomes a more important fact than merely knowing about a person's level of drinking at a single point in time [5]. On the whole, studies examining the consistency of drinking patterns over time are rare [18–21] and even these have typically focused more strongly on finding associations between consumption levels observed at different points in time. Typically what these studies show is that the prediction of adult alcohol consumption from earlier consumption (usually measured during adolescence) can be difficult (see [18–21]). Predicting alcohol consumption at age of 21 years from consumption at 18 years is much easier than predicting consumption levels at age 25 from age 16 levels. This is because not all young people who are drinking at high levels at age 16 necessarily continue to drink this way. Moreover, evidence has emerged in several studies (see [19, 22]) that different patterns may exist. As Wennberg et al. show, there may be some who start low and increase over time, some who decrease over time, and others who consistently drink at a lower or higher level. Another study by Virtanen et al. [23] differentiated between 6 different consumption pathways from age 16 to age 42. In general, their results showed that most of the pathways were very similar (those with generally low-to-moderate consumption levels) and that the strongest association with other risk factors was for those who started drinking at an earlier age (age 16) and who had higher levels of consumption. Such analyses generally do not allow one to examine the effects of consistent differences in dosage or exposure to alcohol over time which is arguably the most common public health interest.

## 2. The Present Study

The aim of this study was to contribute analyses based on recent Australian research that has included measures of alcohol consumption administered to both adolescents and young adults. The data for these analyses are drawn from the South Australian School Leavers project which has tracked several hundred young people from the age of 15-16 years into early adulthood. In this paper, we attempt to identify patterns of lower and higher consumption across time and the extent to which more consistent patterns of elevated drinking can be predicted by sociodemographic and psychological risk factors measured at age of 15-16. Based on our review of the literature, our investigation was designed to examine what risk/background factors appear to be most indicative of the development of consistently high drinking patterns and to explore possible gender differences. Our study compared risk factors in three principal clusters: family and social background including financial wellbeing, psychological wellbeing, and health-related behaviours such as smoking and drug-taking. Our study also controlled for personality differences (most notably extraversion and neuroticism) because these variables have emerged in some studies as correlates of alcohol consumption in previous studies [16]. Mulder's [16] review found that personality explains only a relatively small proportion of variance in alcohol consumption (with evidence of an association with neuroticism) and that delinquent behaviours are stronger predictors. However, extraversion is included in the present study because of its association with stimulus seeking which is often associated with greater risk-taking. In Mulder's view, antisocial or delinquent behaviours appear to be the best predictors. We hypothesised that higher and more consistent patterns of alcohol consumption in early adulthood would be most strongly associated with the following characteristics: (1) male gender, (2) young people with evidence of family dysfunction or disruption, and (3) young people who report internalising problems (lower self-esteem, depression, and anxiety) and other behaviours such as smoking and drug use. Based on Mulder [16], we hypothesised that the predictive relationships would be strongest for this latter class of variables.

## 3. Method

### 3.1. Participants

The data (*n* = 811, *M* = 261, *F* = 548, and 2 missing gender data) for this study were derived from the South Australian School Leavers project and comprised those who provided data for 5 measurement points (from the age of 15 to 20 years of age). The principal outcome measure alcohol consumption was obtained from the 5 measurement points, whereas the predictor variables in this study were taken from the baseline (age 15) survey. This sample was a subset of a larger 10-year longitudinal study which originally involved a baseline sample of 2552 (*M* = 1041, *F* = 1485, and 26 missing gender data) secondary school students with a mean age of 15.2 years (SD = 0.50). Sampling details of this study have been previously described in Delfabbro et al. [24] but are briefly summarised for convenience. Students were recruited from 25 schools in both rural and metropolitan South Australia and this represented just over half of the 45 institutions that had been randomly selected to participate. Participants from metropolitan Adelaide comprised 71.1% (*n* = 1814) of the sample and included participants from 19 schools, whereas 677 (26.5%) students came from rural and regional schools (61 students did not provide their school's name). Matching of the final school sample with population figures showed that the composition of the sample was generally consistent with the government/nongovernment and rural/metropolitan distribution of schools. The data for this study were based on those who remained in the study until Time 5 (age 20) (*M* = 261, *F* = 548).

### 3.2. Procedure

Schools participated with the approval of principals and surveys were conducted under supervision by research assistants and/or teachers. There were no exclusion criteria for participation in the study but all participants were required to obtain parental consent. Since parental approval was required, the eligible population for the study was only those students who took the information sheets home to their parents and sought their consent to participate (between 45 and 70% of all consent forms were returned on the required days across the different schools) which yielded an overall participation rate of 55%. The very strict ethical requirements to obtain parental approval in Australian school research make it very difficult to obtain very high response rates in Australian surveys. Furthermore, when consent forms are not returned, it is unclear whether to classify the case as a genuine nonconsent/refusal or a failure to contact parents. All children who returned their parental consent forms agreed to participate themselves, but 2% of responses had to be discarded due to incomplete or aberrant responding. Conversations with both students and teachers indicated that the failure to return forms was almost solely due to students forgetting to take them home, show them to their parents, or bring them in on the required day. Correlation analysis indicated no evidence that differential return rates across the schools were related to any of the principal variables in the study. The age, gender, and school type (private/government or coeducational versus single-sex composition) did not differ significantly from the state as a whole.

### 3.3. Sample Attrition

The attrition rate for this study was commensurate with a similar and highly cited school leavers study conducted by Winefield et al. [25]. Around 40% of the sample was lost from age of 15 to 16 years, but sample size was more consistently maintained thereafter. By Time 5, 811 of the sample still remained in the study. Attrition analysis conducted by Delfabbro et al. [26] showed that males were more likely to leave the study than females: at Time 5, 67% of the sample were female versus 59% at Time 1 [26]. Missing data was MAR (or missing at random) which indicated that other known variables in the dataset enable one to predict which cases are missing. Multiple imputation methods conducted by Delfabbro et al. [26] revealed that imputing missing values for the principal psychosocial measures led to only trivial changes in the distribution of scores. Moreover, comparisons of those who were retained or not retained until Time 5 showed that the two groups were matched with respect to self-reported alcohol consumption (both frequency and total consumption at Time 1) but differed at the other time points. In other words, those who drank more alcohol were less likely to be retained in the study. This does not invalidate comparisons between those engaged in higher and lower levels of consumption across time but means that the variability between these classifications is diminished. In other words, the percentile classifications in this study capture fewer and less heavy drinkers than should be the case in the actual population of students. This form of attrition is difficult to avoid in studies of this kind. Even in studies with very high retention rates (e.g., 95%+) (e.g., [27]), it has been found that students who drink at higher levels are significantly more difficult to retain in the sample.

*Measures*

*(i) Demographic/Background Variables*. Participants were asked to report their gender, age, and ethnicity/cultural identity (Aboriginal or Torres-Strait Islander descent). They were also asked whether their parents cohabitated (Yes/No) and whether there was any unemployment in their immediate family. Participants also completed a Financial Security Scale [25]. Participants were required to rate their agreement with twelve statements according to a 4-point scale (1 = Strongly agree; 4 = Strongly disagree). Example items included the following: “I have enough money to meet my personal needs” and “I am under strain as far as money goes.” Reliability analysis of this measure indicated that it had an alpha of 0.86.

*(ii) Health-Related Variables*. Participants were asked to give a rating of their health over the last 12 months (1 = Very healthy most of the time; 5 = Nearly always ill) and this was rescored into a binary variable (1 = Healthy; 2 = Unhealthy) by classifying scores of 3 or higher as being indicative of at least moderate good health and scores of 4-5 as indicating poorer health. A second variable asked participants to indicate their weight status (1 = Very underweight to 5 = Very overweight, with 3 = Normal weight). This variable was also rescored into a binary variable (Overweight and Other) to allow comparisons of overweight individuals with those who perceived themselves to be of normal weight or underweight. A third question asked respondents whether they had any physical health conditions (Yes/No). Involvement in high risk activities was assessed by asking students whether they smoked (Yes/No), drank alcohol (1 = Yes; 2 = No), and used marijuana (Yes/No) or other harder drugs (Yes/No). For the alcohol question, participants indicated how frequently they drank with a scale that ranged from 1 = < Drank once per year to 5 = Daily.

*(iii) Social Variables*

*(a) Victimization/Bullying*. Participants were asked to report their experiences of being bullied by peers at school and outside of school in the last 12 months. Participants were presented with five statements regarding various forms of victimization (i.e., “I get picked on by other kids,” “I get picked on by some teachers,” “I get hit and pushed around by other kids,” “Other kids make fun of me,” and “I get called names by other kids”) and were asked to rate on a 4-point scale the extent to which each of these had been their experience both in school and outside of school (1 = Never; 4 = Very often). The 8 items were combined to yield a total bullied-by-peer score that could range from 8 to 32 (maximum). The bullying-by-peers scale was found to have very good internal reliability (*α* = 0.82).

*(b) Peer Relationships*. Participants were asked to report the number of friends they currently had (a number between 0 and 30) and the number of their class peers that they do not like (0–30) and to estimate the number of class peers that disliked them (0–30).

*(c) Family Functioning*. The General Functioning Scale from the Family Assessment Device [28] was used as a measure of family functioning (11 of 12 items were included because one item reduced the alpha value of the scale: “We cannot talk to each other about the sadness we feel”). The items selected for the present study all provided global measures of family functioning (problem solving, communication, roles, affective responsiveness, affective involvement, and behavioural control). Participants were asked to rate the extent to which each aspect of family functioning currently described their immediate family on a scale of one to four (1 = Strongly agree; 4 = Strongly disagree) giving a score range of 11 to 44 (excellent functioning). This measure proved to have good internal consistency, *α* = 0.77.

*(iv) Psychological Variables*

*(a) Self-Esteem*. Self-esteem was measured using Rosenberg's [29] self-esteem scale. This scale consists of 10 items and respondents indicate their current level of agreement (1 = Strongly agree, 2 = Agree, 3 = Disagree, and 4 = Strongly disagree). The scoring range was from 10 (low self-esteem) to 40 (high self-esteem). This scale had very good internal reliability in the present sample, *α* = 0.82.

*(b) Psychological Health*. The General Health Questionnaire (GHQ-12 [30]) was designed as a screening instrument to provide information on current mental wellbeing in community samples, rather than giving specific psychiatric diagnoses. The General Health Questionnaire 12 is a shortened version of the original 60-item questionnaire that was developed to detect minor psychiatric illness in community populations. The GHQ-12 provides a list of twelve symptoms. Participants were asked to rate the degree to which they had experienced each symptom in the past few weeks by selecting one of four response categories (i.e., 1 = More so than usual, 2 = Same as usual, 3 = Less than usual, and 4 = Much less than usual). The scores were recorded using the standard binary coding method (0,0, 1,1) in which “symptomatic responses” were scored as a one. This method of scoring resulted in scores that ranged from 0 to 12 with higher scores signifying more psychological distress. The GHQ manual notes that the scale can be used with adolescents. The internal reliability was very good, *α* = 0.80.

*(c) Social Alienation*. The Dodder and Astle [31] social alienation scale consisted of nine statements and respondents were required to indicate whether they currently agreed or disagreed with each statement. This scale was adapted from Srole's Anomie scale [32] designed to measure a person's perception of meaninglessness in society, or cynicism. This measure had a barely acceptable internal consistency, *α* = 0.60.

*(d) Life-Satisfaction*. An abbreviated version of Warr et al.'s [33] life-satisfaction scale was administered. This consisted of 7 items describing aspects of life (e.g., education, family life, and present government) and respondents indicated their current level of dissatisfaction on a 5-point scale ranging from 1 = Extremely dissatisfied to 5 = Extremely satisfied. Possible scores ranged from 7 (Low satisfaction) to 35 (High satisfaction). This scale had acceptable internal reliability, *α* = 0.73.

*(v) Personality Measures*. Participants completed the Costa Jr. and McCrae [34] extraversion and neuroticism subscales. Each scale comprises 12 items which are answered without any time-scale using a 5-point Likert scale ranging from 1 = Strongly disagree to 5 = Strongly agree to yield a scoring range of 12–60. Both of these scales had very good internal consistency in this sample, both alphas: *α* > 0.80.

### 3.4. Alcohol Consumption

Alcohol consumption levels were calculated using a similar method to that adopted by Janlert and Hammarström [35] in other longitudinal research. At each time point, the estimated total number of standard drinks per year was calculated by multiplying the reported frequency of alcohol consumption by the typical amount reported being consumed on each occasion. This total was then used to divide males and females into higher versus lower consumption groups based on percentile split: those above the 75th percentile for this sample were classified as higher for that particular year. This yielded a high/low division over five consecutive years for both genders. This method was used because it is likely to be of greater value in public policy contexts than very abstract trajectory and latent class models which develop groupings which cannot easily be translated to prevalence data in the public domain. Current National Health and Medical Research Council (NHMRC) statistics indicate that around 30% of Australians aged 14 years and older report single binge-drinking episodes likely to be harmful to health and that 20% report chronic risk. Given these figures and the focus on early adult drinking in this study, the upper 25% of the drinking distribution appeared to be sensible group upon which to focus so as to maximise the value of the research to Australian policy-makers.

Some indicative drinking statistics are provided to indicate the extent to which the percentiles differentiated respondents (Table 1). This table shows the estimated number of self-reported standard drinks per year for both men and women as well as for the sample as a whole. These figures were obtained by multiplying the reports numbers of drinking sessions per year by the typical number of drinks reported per occasion. In Australia, under the guidelines released by the National Health and Medical Research Council (NHMRC) [36], safe drinking levels are said to be exceeded when people consume two or more standard drinks per day (or around 730 drinks per year). As indicated in Table 1, the lower group clearly remained well below this threshold across the 5 years, whereas the higher group had exceeded this level by Time 4 and was maintaining a level corresponding to more than 1.5 standard drinks per day at Time 5. These levels were not observed for women, so that the overall figures reflect the more elevated rates observed in men. Given the finding that actual drinking levels are likely to be higher than self-reported levels (see [37]), we were confident that our high group characterised a group of people who were drinking at a level likely to have longer-term drinking consequences.

Yearly percentile classifications were then used to establish two groups that differentiated between consistently higher and lower levels of consumption: (1) low consumption (the respondent was consistently below the 75th percentile in all 5 years) and higher: the respondent was in the upper percentile group in 3–5 of the years. For males, there were 127 cases in the lower group and 41 in the mostly high group. For females, the two groups had 418 and 45 cases.

Bivariate analyses (chi-squared and *t*-tests) were used to analyse the principal outcome variables associated with membership in the two groups. The modelling used several steps. First, to examine the value of running separate gender-specific models, the analysis commenced with a test of gender interactions for each of the predictor variables. Predictors were standardised and centred and product terms were created with gender and then entered into logistic models after controlling for the main effects of the two separate variables. Given that these results yielded no significant interactions, an overall model was developed. A second step of the analysis involved testing for the presence of any significant multicollinearity. Following the procedures set out by Midi et al. [38], this analysis produced variance inflation factors (VIFs) and tolerance estimates for each of the potential predictors. The results showed that no VIF exceeded 2.5. Thus, while a number of predictors were moderately correlated, these relationships did not create a level of multicollinearity that would be considered excessive or problematic. The third step involved entry of the variables into the equation. Modelling was conducted using backwards elimination and the log-linear likelihood ratio as the test statistic to differentiate between models.

An initial model included all variables that were found to differ across consumption groups for either men or women. This showed that none of the variables that were significant for one gender only made statistically significant contributions to the model. A second model was then run only using those variables where differences were observed across both genders. This is conceptually more robust in that it avoids a potential fallacy of composition by basing the model on predictors that might be only significant only for one of the genders. Given the similarity between the male and female models, a final model was developed which examined the strongest predictors of group membership after controlling for gender.

All of these analyses were performed using PASW-v.20 after having used the R-package (v.2.14.1) to test for any evidence of clustering effects due to the sample having been drawn from schools. A variance components analysis of a base intercept model showed that only a trivial amount of variance could be accounted for by school membership. The intraclass correlation (ICC) which is based on the ratio of the between-cluster variation and total variation was found to be between 0.01 and 0.03 in all models tested. We found trivial differences between the models run using school as a nesting factor as compared with those conducted without it. Given this finding and the fact that two waves of data occurred after leaving school, we have presented standard models which are generally easier to interpret than mixed models. It should also be noted that observations were also not statistically nested within cases because longitudinal data was used to develop a between-groups design rather than one that examined the grouping effect in each year in a repeated measures design. Here the focus was on usage patterns over 5 years.

## 4. Results

### 4.1. Bivariate Analyses

Inspection of the results for men (Table 2) showed that those in the higher group were generally similar in terms of demographics, although they were more likely to come from families which were experiencing unemployment. This group was more likely to report smoking and marijuana use during adolescence, poorer family functioning, and poorer scores on all measures of psychological wellbeing. The results for women were generally very similar in terms of the psychological wellbeing and substance use variables, but there were some differences. Women who drank at higher levels were more likely to come from families where parents had separated and where there was less financial security. Women in this group were also more likely to report higher neuroticism scores at age 15 years and were less popular with their classmates.

### 4.2. Logistic Regression

The logistic regression (Table 3) showed that the strongest predictors of higher drinking levels during early adulthood were gender (males were four times more likely to fall into this group). Those who smoked at age 15 were 8 times more likely to drink at higher levels, whereas using marijuana was associated with almost a threefold increase in risk. Having poorer general health scores was also significantly associated with higher risk along with poorer family functioning (although this effect marginally failed to reach significance).

## 5. Discussion

The aim of this paper was to identify the adolescent social and psychological factors that are associated with elevated drinking patterns during early adulthood. The study investigated several hypotheses and a number of these were generally confirmed. Higher levels of alcohol consumption in early adulthood (often above the NHMRC guidelines) were associated with male gender. Young adults who drink more tend to have a history of engaging in other activities that are potentially detrimental to their long-term health, including smoking and using drugs. They are also more likely to report poorer peer relationships and poorer psychological functioning in the form of lower life-satisfaction and lower self-reported general health (as measured by the GHQ-12). Our results also showed that these patterns were generally consistent across both genders and were consistent with the review undertaken by Nolen-Hoeksema [17] and more recent research conducted by Dubow et al. [39] and Marmorstein [14]. Despite some subtle variations in the univariate differences observed for each gender separately, multivariate analyses found that final models were very similar for both genders and that an overall model could be presented. This showed that adolescent smoking, marijuana use, and psychological wellbeing were found to be the strongest predictors after other factors such as gender had been controlled.

The relationships observed for peer variables were generally inconsistent. Although having poorer relationships with other peers at school was generally related to higher levels of drinking for young women, those in the higher drinking group did not necessarily report the lowest number of close friends or a disconnection from others. For example, it was young women who reported having more close friends during adolescence who reported drinking more during early adulthood. These inconsistencies have been discussed by Scholte et al. [6] who argue that the role of social relationships in adolescent drinking is likely to be complex. In their view, drinking probably needs to be interpreted in its social context. In some schools or environments, where few peers drink, it may be that drinking is considered a sign of confidence and maturity. In this sense, the influence of drinking behaviour amongst peers may vary developmentally over time. For other young people, it may be symptomatic of other behaviours that reflect a rejection of parental norms and societal expectations. At the time, even when there is deviance in attitude, young people who form part of social groups who drink more heavily may gain a sense of affirmation and solidarity through this activity. Thus, while such young people may not be popular with other peers, they may have many friends who share the same attitudes and behaviours. These results are generally consistent with Scholte et al.'s observations that heavy drinking is not always inimical to successful social relationships, but the findings support the view that more attention needs to be directed towards understanding the relationship between drinking and the broader social structure of peer groups. As our results indicate, asking whether a person has social connections alone may not capture the fact that this person belongs to a social subgroup that might be less well connected or liked by other peers.

A number of factors should, however, be taken into account when interpreting these findings. The first is that the results were obtained only in one social context (Australian secondary schools) so it is unclear whether the findings can be generalised to other countries where there may be a lower prevalence of drinking amongst young people and different social norms relating to drinking. Second, this study only examines drinking patterns into early adulthood, so it is not clear whether these risk factors will continue to predict longer-term drinking patterns that might have health consequences. As Wennberg et al. [22] have cautioned, although it is useful to study multiple time points when studying alcohol consumption patterns, there may be more complex patterns that are not detected using the strategies which we have used. For example, while our higher and lower groups usefully differentiate between different levels that are maintained over an extended period, it would be possible in larger studies to examine other patterns, for example, those who start at lower levels and increase over time or those who show the reverse pattern. A third consideration is that our study (because of the modest size of the sample) focuses only on statistically elevated drinking levels but does not examine the predictors of binge levels of consumption. Thus, while these findings are of interest in understanding the consistency of elevated drinking patterns, the findings cannot be compared with studies that focused on problematic levels of drinking. Fourth, although our analysis of sample attrition does not indicate any strong threats to the validity of these findings, we would emphasise that some more nuanced analyses may have been possible if a larger proportion of the baseline sample had been maintained over time. Fifth, in conducting gender differences across a variety of measures, we assume that measurement properties for these covariates are the same for each gender. Finally, although this study measured some variables (e.g., marijuana and cigarette smoking) as indicators of cross-substance use, the study did not formally assess conduct disorder or externalising behaviour which are known to be strongly associated with adolescent substance abuse.

In conclusion, our study provides support for the view that early substance use in adolescence is the strongest indicator of future alcohol use in early adulthood, even after controlling for psychological and other family background factors. These patterns were observed for both males and females, although we observed that the stability of family environments may play a more important role for young women. In future analyses, we hope to extend these analyses to examine to what extent different levels of alcohol exposure contribute to changes in indices of psychological and social wellbeing over time.

## Figures and Tables

**Table 1:** Mean (SD) standard drinks for the low and high consumption groups across 5 years.

	Year 1 M (SD)	Year 2 M (SD)	Year 3 M (SD)	Year 4 M (SD)	Year 5 M (SD)
*Men*					
Lower	81.2 (635.6)	109.0 (348.21)	215.9 (593.53)	372.4 (998.20)	315.4 (658.88)
Higher	305.4 (11.07)	346.7 (13.8)	746.0 (38.1)	1165.5 (116.7)	962.2 (152.8)

*Women*					
Lower	53.6 (339.93)	64.2 (298.41)	120.0 (280.16)	185.8 (360.19)	198.1 (357.46)
Higher	448.5 (11.80)	237.3 (103.76)	239.5 (198.68)	288.9 (312.53)	341.9 (332.19)

Overall	61.2 (505.04)	326.8 (75.91)	530.5 (145.39)	865.7 (235.62)	606.3 (229.30)

**Table 2:** Comparative characteristics of alcohol consumption groups.

	Lower consumption		Higher consumption		Alcohol group comparisons		
	Men *N* (%)	Women *N* (%)	Men *N* (%)	Women *N* (%)	Men *X* ^2^(1)	Women *X* ^2^(1)	Overall *X* ^2^(1)
*Demographics*							
Parents live together (Yes)	108 (85.0)	331 (80.9)	34 (82.9)	27 (61.4)	<1	9.18^*∗∗∗*^	4.81^*∗*^
Family unemployment (Yes)	26 (20.8)	87 (21.6)	3 (7.3)	10 (22.2)	3.89^*∗*^	<1	1.81
Aboriginal (Yes)	a	12 (2.9)	a	0 (0.0)	a	1.36	a

	M (SD)	M (SD)	M (SD)	M (SD)	*t*(162)	*t*(415)	*t*(577)

Financial wellbeing scale	34.9 (6.06)	34.2 (4.15)	34.7 (5.0)	32.2 (5.76)	<1	2.49^*∗*^	1.67

	*N* (%)	*N* (%)	*N* (%)	*N* (%)	*X* ^2^(1)	*X* ^2^(1)	

*Health-related behaviours*							
Heath poor	a	11 (2.7)	a	0 (0.0)	a	1.21	<1
Overweight	19 (15.1)	77 (18.8)	8 (19.5)	12 (26.7)	<1	1.58	1.38
Physical health problems (Yes)	25 (19.8)	73 (18.0)	10 (24.4)	10 (22.2)	<1	<1	1.10
Smoker (Yes)	6 (4.8)	18 (4.4)	8 (19.5)	18 (40.0)	8.76^*∗∗∗*^	70.17^*∗∗∗*^	66.18^*∗∗∗*^
Marijuana (Yes)	5 (4.0)	7 (1.7)	9 (22.0)	13 (28.9)	13.03^*∗∗∗*^	71.10^*∗∗∗*^	77.98^*∗∗∗*^

	M (SD)	M (SD)	M (SD)	M (SD)	*t*(162)	*t*(415)	*t*(577)

*Personality*							
Extraversion	43.3 (6.56)	43.4 (6.19)	41.5 (5.95)	42.4 (6.09)	<1	<1	1.81
Neuroticism	30.3 (7.02)	34.4 (7.92)	31.9 (5.36)	38.3 (8.74)	1.33	2.96	1.88

*Family and peer relations*							
Family functioning	33.9 (5.76)	32.6 (6.62)	31.5 (6.96)	29.6 (7.35)	2.05^*∗*^	2.72^*∗*^	2.96^*∗*^
Number of close friends	12.7 (8.36)	11.8 (7.85)	14.6 (9.54)	13.1 (1.31)	1.56	<1	1.86
Kids not liked	5.2 (5.84)	4.4 (5.03)	6.3 (6.19)	5.0 (5.38)	<1	<1	1.69
Kids not like you	4.0 (4.96)	3.3 (4.94)	4.7 (4.91)	5.7 (5.88)	<1	2.42^*∗*^	2.56^*∗*^
Bullied by peers	6.4 (2.55)	5.6 (1.87)	6.33 (2.34)	5.8 (2.28)	<1	<1	1.11

*Psychological wellbeing*							
Self-esteem	33.6 (4.80)	30.3 (5.33)	31.4 (4.07)	27.4 (5.74)	2.53^*∗*^	3.40^*∗∗*^	2.86^*∗*^
GHQ-12	1.6 (1.82)	3.0 (2.49)	2.9 (2.23)	4.2 (3.25)	3.58^*∗∗*^	2.42^*∗*^	2.70^*∗*^
Social alienation	3.5 (2.13)	3.3 (2.03)	4.7 (2.18)	4.0 (1.95)	3.06^*∗∗*^	2.07^*∗*^	3.95^*∗∗∗*^
Life-satisfaction	26.9 (3.81)	26.4 (4.03)	24.6 (4.39)	24.2 (4.44)	3.21^*∗∗*^	3.18^*∗∗*^	4.27^*∗∗∗*^

^*∗*^ *p* < 0.05, ^*∗∗*^ *p* < 0.01, and  ^*∗∗∗*^ *p* < 0.001; male lower group (*n* = 124–127); male higher group (*n* = 39–41); female lower group (*n* = 380–409); female higher group (*n* = 41–45); a = sample size too small to allow valid analysis.

**Table 3:** Logistic regression analysis: significant age 15 predictors of drinking level over 5 years.

	*B*	SE	Wald	OR	95% conf int
Intercept	−2.09				
Family adjustment	−0.04	0.02	3.61	0.96	0.92–1.00
Smoking	2.06	0.42	24.63^*∗∗∗*^	7.87	3.48–17.76
Marijuana use	1.08	0.54	4.02^*∗*^	2.95	1.03–8.49
GHQ-12	0.16	0.06	7.64^*∗∗*^	1.17	1.05–1.30

^*∗*^ *p* < 0.05, ^*∗∗*^ *p* < 0.01, and ^*∗∗∗*^ *p* < 0.001.

Notes: dependent measure coded (1 = lower drinking level; 2 = higher drinking level). Self-esteem, social alienation, and life-satisfaction were eliminated because their inclusion did not contribute to any significant changes in the log-linear likelihood ratio.
